# Identification of key factors for early detection of rheumatoid arthritis in primary care using machine learning

**DOI:** 10.1038/s41598-025-34158-1

**Published:** 2026-01-12

**Authors:** Fatemeh Rahimi, Elham Rajaei, Noushin Movafagh, Ali Mohammad Hadianfard

**Affiliations:** 1 Department of Health Information Technology, School of Allied Medical Sciences, Ahvaz Jundishapur University of Medical Sciences, Ahvaz, Iran; 2 Golestan Hospital Clinical Research Development Unit, Ahvaz Jundishapur University of Medical Sciences, Ahvaz, Iran; 3 Department of Rheumatology, School of Medicine, Ahvaz Jundishapur University of Medical Sciences, Ahvaz, Iran; 4 Department of Health Information Technology, School of Allied Medical Science, Ahvaz Jundishapur University of Medical Sciences, Ahvaz, Iran

**Keywords:** Rheumatoid arthritis, Primary health care, Artificial intelligence, Machine learning algorithms, Early diagnosis, Diseases, Medical research, Rheumatology

## Abstract

Rheumatoid arthritis (RA) is a chronic disease that causes irreversible joint damage. Early detection, especially in primary care settings, is crucial for effective disease management. This study aimed to identify the factors that help screen individuals at risk of RA to reduce delays in referral to rheumatologists. This analytical and applied research used a questionnaire to gather data from 377 patients at a rheumatology diagnostic center in Ahvaz, Iran, between August and November 2024. Study variables included patients’ articular and extra-articular symptoms at disease onset, demographic data, and initial laboratory markers. After performing statistical correlation analysis, the dataset was split into training (80%) and testing (20%) subsets. Five machine learning models were developed, and the SHAP method was applied to the best-performing model to identify influential features. The results were obtained via 5-fold nested cross-validation, which identified the CatBoost model as the top performer, with AUC-ROC = 0.966, Accuracy = 0.947, and F1-Score = 0.951. SHAP (with a threshold of 0.01) highlighted the following significant features: Anti-CCP, tender joint count, swollen joint count, gastrointestinal issues, fatigue, age, RF (Rheumatoid Factor), and hearing problems. Due to the importance of early RA diagnosis and the challenges encountered in primary care, three main screening factors stand out: Anti-CCP, tender joint count, and swollen joint count. These, along with fatigue, age, and positive RF, markedly increase the likelihood of RA and justify referring a patient to a specialist.

## Introduction

Rheumatoid arthritis (RA) is a chronic, debilitating autoimmune disease caused by immune dysfunction^1^. It is among the most common autoimmune disorders^2^. In RA, the synovial joints are attacked by the immune system, leading to inflammation and irreversible damage^3^. This joint damage results in functional disability, reduced quality of life, increased risk of comorbidities, financial burdens, and even premature death^4^. Early referral of individuals with suspected joint inflammation to rheumatologists plays a crucial role in facilitating timely diagnosis and effective disease management^5^. National and international guidelines emphasize the importance of rapid referral, yet studies show that only 17% of patients receive timely and accurate referrals^6^.

The 2010 American College of Rheumatology (ACR)/European League Against Rheumatism (EULAR) classification criteria for RA, while helpful for rheumatologists, have low specificity^7^ and cannot diagnose RA in all cases^8^. The diversity of extra-articular symptoms, overlap with other diseases^4^, the transient and ambiguous nature of early joint symptoms^3^, and the lack of a definitive diagnostic laboratory test or imaging technique make identifying at-risk individuals—particularly for primary care physicians—challenging. Consequently, referrals for suspected RA and access to specialized care are often delayed^6^. Given the potential of machine learning (ML) algorithms as advanced analytical tools to enhance the efficacy of medical care^4^, these methods can be leveraged. ML, a subset of artificial intelligence (AI), is a powerful analytical approach designed for automated data learning and decision-making^9^.

Although ML applications in rheumatology are relatively recent compared to other fields, studies have explored risk assessment, disease diagnosis, patient phenotyping using Electronic Health Records (EHR) or Electronic Medical Records (EMR), treatment response prediction, disease progression, comorbidity risk, and biomarker discovery^10^. Most ML-based RA diagnostic studies have used joint images from X-rays or Magnetic Resonance Imaging (MRI), while fewer have focused on clinical data. However, the most accessible resource for primary care physicians is patient-reported symptoms^11^.

Factors such as post-rest pain, morning stiffness, symmetric joint involvement, body pain, joint redness/swelling, age, sex, prior joint damage, and laboratory markers like Anti-cyclic.

Citrullinated peptides (Anti-CCP), Erythrocyte Sedimentation Rate (ESR), Rheumatoid Factor (RF), and Anti-Carbamylated Protein (Anti-CarP) have been used in previous modeling studies^5,6,8,12,13^.

This study aimed to identify the effective factors for early RA detection in primary care by modeling demographic, articular, and extra-articular symptoms, along with laboratory data, using machine learning algorithms and feature selection methods. Additionally, it sought to determine the most important disease-related features.

## Methods

This study was an analytical-applied research. The study population included patients who visited a rheumatology diagnostic center in Ahvaz between July and October 2024. To determine the sample size, considering that two groups—patients and non-patients (healthy individuals)—were examined in this study, Cochran’s formula was used. A total of 377 participants were selected from the center’s visitors and divided into two groups: patients diagnosed with rheumatoid arthritis (RA) by a rheumatologist and receiving treatment, and a control group consisting of individuals who exhibited symptoms but were not diagnosed with RA.

To be included in the study, participants had to meet specific criteria: their RA diagnosis had to be confirmed by a rheumatology subspecialist, and they had to be 18 years or older. Exclusion criteria included active bacterial or viral infections, crystalline arthropathies, vasculitis, malignancies, pregnancy, or lactation.

Data were collected using a structured questionnaire that covered 40 variables, including clinical symptoms, demographic information, and laboratory findings at the time of disease onset.

The questionnaire’s validity was assessed and confirmed by two rheumatologists, and its reliability was tested through a test-retest method with 15 patients, resulting in a high.

reliability coefficient of 92%. The statistical methods initially used to analyze the data and examine their correlation with the target variable, depending on the data type, were Spearman’s rank correlation and the Chi-square test. Based on the correlation coefficient and p-value, factors with a significant relationship to the target variable were identified.

Next, the dataset was split into 80% for training and 20% for testing. Machine learning algorithms and modeling techniques were then applied to disease detection, including Logistic Regression, Lasso Regression, and three decision tree-based models: Random Forest, Extreme Gradient Boosting (XGBoost), and Categorical Boosting (CatBoost).

Logistic regression, introduced by Cox in 1958^14^, is a classification method in supervised machine learning that uses the odds ratio to analyze relationships between variables. The term “Lasso” **(**which means *“rope "* in Spanish) refers to a regression

that, true to its name, aims to “lasso” (select) the most relevant variables. In contrast, the method discards the less important ones, thereby producing a simpler and more interpretable model^15^.

Random Forest is an ensemble learning algorithm that combines multiple decision trees to improve predictive accuracy^16^. The XGBoost algorithm is an advanced and more sophisticated version of the Random Forest algorithm^17^. Renowned for its speed, efficiency, and high accuracy, XGBoost leverages specialized optimization techniques to deliver exceptional performance even in complex problem domains. CatBoost is a robust machine learning algorithm specifically designed to handle categorical data efficiently^18^. Built upon gradient boosting principles, it requires minimal data preprocessing, making it highly practical for real-world datasets with mixed feature types. TabNet, a state-of-the-art transformer-based model^19^, was employed in this study due to its modern and powerful architecture. However, its performance was suboptimal—likely due to the limited sample size—and it was consequently excluded from the final model comparison. After implementing the models, their performance was rigorously assessed using the AUC-ROC (Area Under the Receiver Operating Characteristic Curve), Accuracy, and F1-Score in a 5-fold nested cross-validation and Calibration Plot Analysis. Consequently, the best model was selected as the final model for further application. To enhance the transparency and interpretability of the predictive models, we employed SHAP (Shapley additive explanations)^20^, a mathematically rigorous framework based on cooperative game theory, to quantify each input feature’s contribution to the model’s predictions^21^. Following the identification of key predictive factors, the selected CatBoost model was retrained using only the most influential variables (as determined by SHAP analysis). Its performance was then rigorously re-evaluated through 5-fold cross-validation and compared with the original baseline model.

In general, machine learning studies lack a specific formula or standard method for determining sample size, and large sample sizes are typically recommended^22^. However, in medical research, particularly when dealing with real-world data collected at the patient’s bedside, obtaining a large sample size is often not feasible due to existing constraints. Consequently, a post hoc power analysis was conducted to assess the statistical power of the sample size.

### Ethical statement

Although this study was not classified as a traditional interventional or clinical trial, it involved human data. Therefore, all research methods were carefully conducted in accordance with relevant guidelines and regulations that govern such studies. It is important to note that informed consent was obtained from all participants. A copy of the consent form in Persian is available. Additionally, to ensure ethical compliance, the study protocol was thoroughly reviewed and approved by the Ethics Committee of Ahvaz University of Medical Sciences with reference number IR.AJUMS.REC.1403.181. This approval highlights the commitment to uphold the highest ethical standards in research involving human subjects.

## Results

The study sample comprised 377 participants with a mean age of 41.93 ± 1.3 years (95% CI). It consisted of 350 females (92.8%) and 27 males (7.2%). The marital status distribution of participants showed 337 married (89.4%) and 40 single (10.6%). Additionally, the participants’ educational status included 200 individuals with less than a high school education (53.1%), 99 high school graduates (26.3%), 61 with university degrees (16.2%), and 13 with postgraduate qualifications (3.4%). The sample consisted of 54% patients and 46% controls, all of whom were analyzed using statistical and machine learning methods. The results of statistical tests (Spearman’s test for non-normal numerical and ordinal variables, and the chi-square test for categorical variables) showed that Anti-CCP, the number of tender joints on examination, and the number of swollen joints were strongly correlated with the target variable (disease status). Additionally, RF and ESR showed a moderate correlation with the target variable. The remaining variables did not show a significant association or meaningful correlation with the target variable. (Table 1)

**Table 1:** Significant features in spearman’s and Chi-Square statistical Tests.

	Features	Description	Statistical test	Correlation Coefficient	Significant
1	anti_ccp	Biomarker	Spearman	0.696	Yes
2	tender_j_c	Tender joint count upon clinical examination	Spearman	0.690	Yes
3	swollen_j_c	Swollen joint count	Spearman	0.620	Yes
4	Rf	Biomarker	Spearman	0.489	Yes
5	Esr	Biomarker	Spearman	0.336	Yes

The correlation coefficient ranges from − 1 (perfect inverse correlation) to + 1 (perfect positive correlation). A value of 0 indicates no correlation. Values below 0.39 represent weak correlation, 0.40–0.59 indicate moderate correlation, 0.60–0.79 signify strong correlation, and 0.80–1 denote very strong correlation.

Subsequently, machine learning algorithms were employed for data mining. Five models— Logistic Regression, Lasso Cross-Validation Regression, Random Forest, XGBoost,

and CatBoost—were applied to the dataset to evaluate their performance in disease identification. To optimize the hyperparameters of tree-based models, a grid search method was employed.

The models’ performance was further evaluated using 5-fold nested cross-validation, with the results presented in Table 2.

**Table 2:** Performance comparison of the machine learning models using 5-fold nested cross-validation.

	Model	AUC- ROC	Accuracy	F1- Score	Log loss	Brier Score	Sensitivity	Specificity
1	CatBoost	0.966	0.947	0.951	0.217	0.060	0.951	0.943
2	Random Forest	0.967	0.908	0.916	0.282	0.078	0.927	0.886
3	Lasso Regression	0.965	0.895	0.900	0.254	0.074	0.878	0.914
4	XGBoost	0.955	0.921	0.927	0.246	0.065	0.927	0.914
5	Logistic Regression	0.941	0.829	0.847	0.361	0.114	0.878	0.771

Although the Random Forest model achieved the highest AUC-ROC (0.967), the CatBoost model.

was selected as the final model due to its superior and more balanced performance across other key.

metrics, including Accuracy, F1-Score, Sensitivity, and Specificity, as well as its better-calibrated predictions, evidenced by lower Log Loss and Brier Score values.

The SHAP method was then applied to the CatBoost model to determine feature importance,

with the results presented in Table 3.

**Fig. 1:**
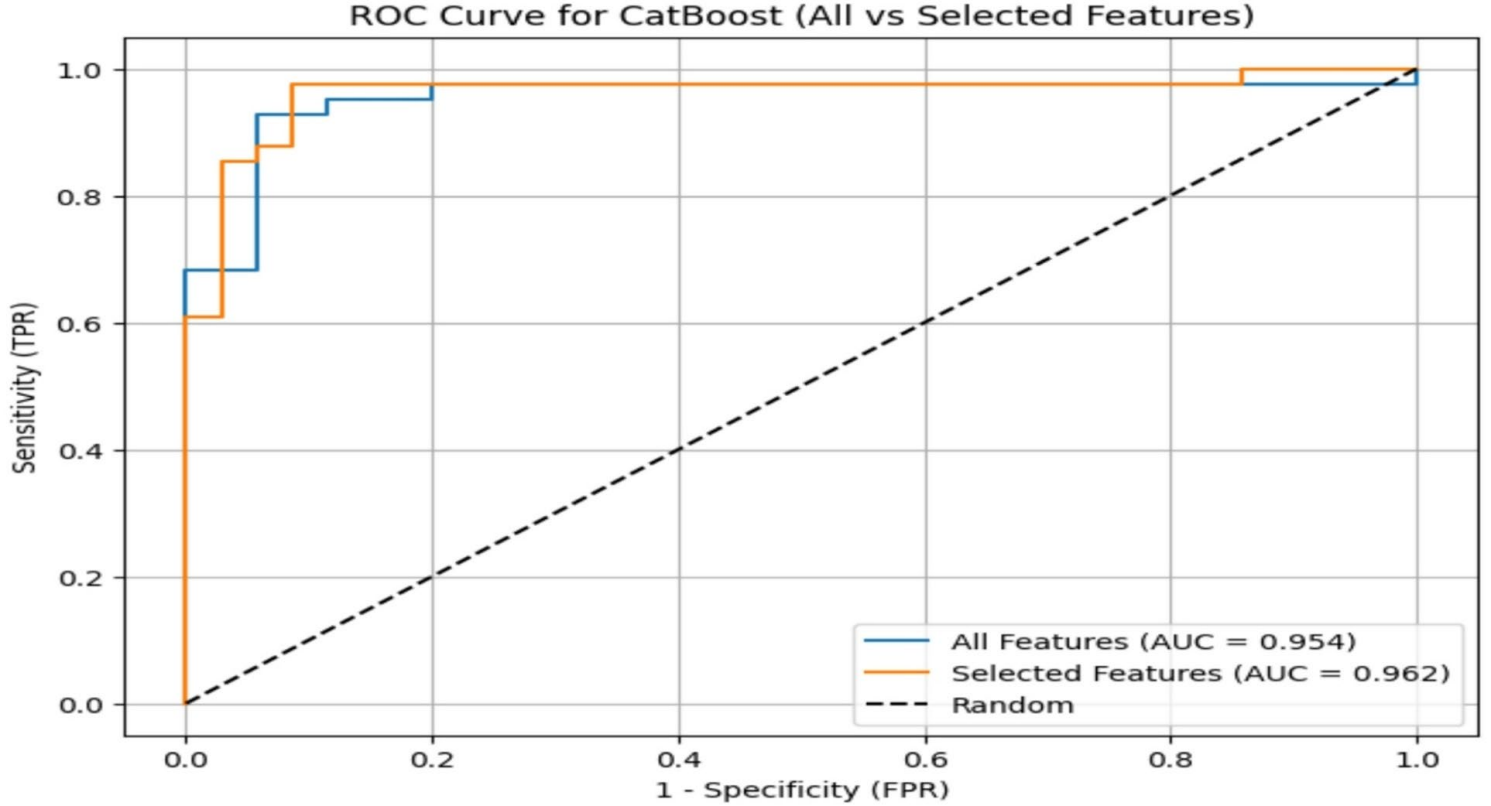
ROC Curves for the CatBoost model: baseline features vs. selected features.

**Fig. 2:**
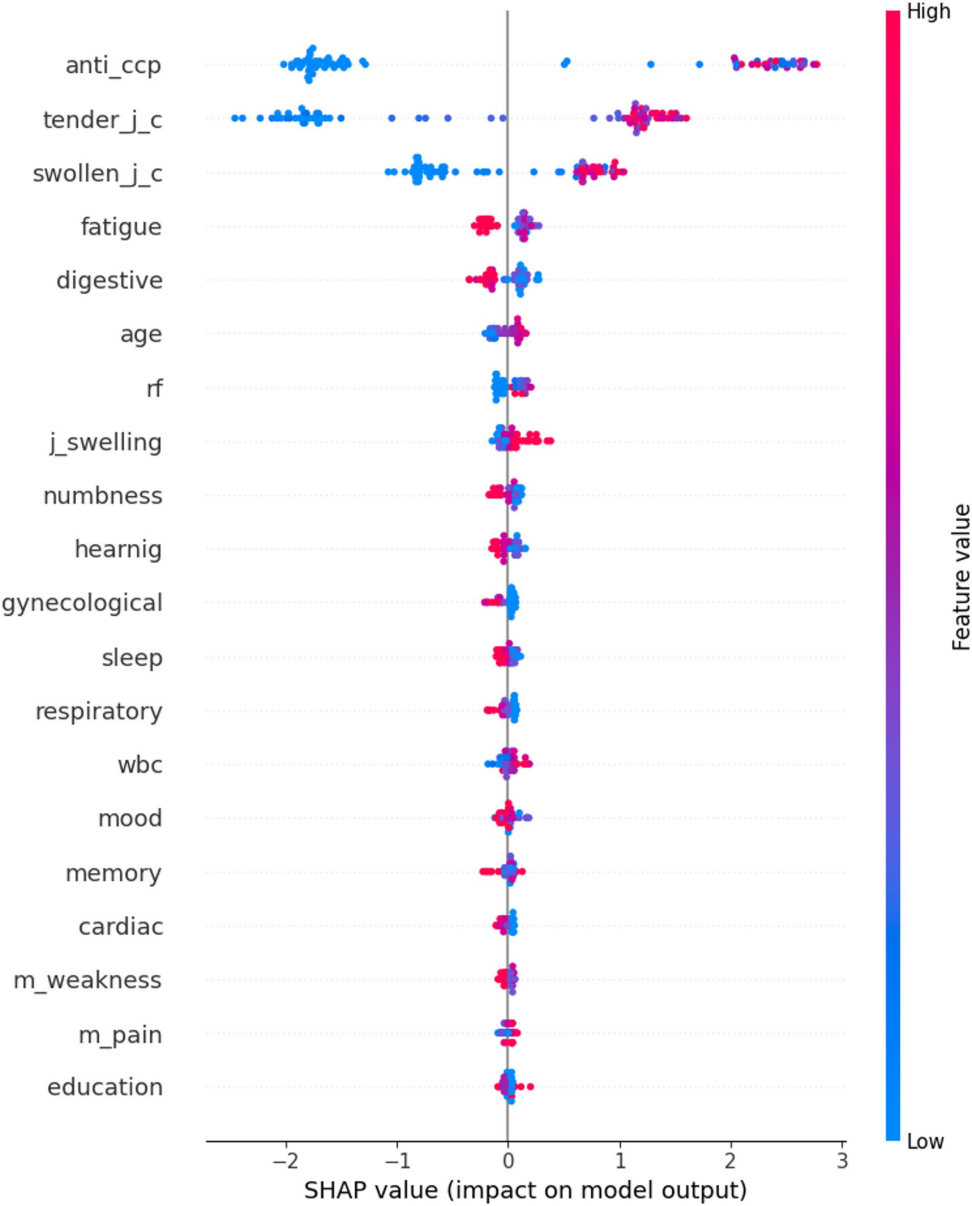
SHAP Summary Plot.

Based on the post-hoc analysis results, the statistical power and effect sizes of the study variables indicated that the most important features identified by the SHAP technique had the highest statistical power and substantial effect sizes. This indicates both high reliability and significant predictive impact of these features in disease prediction (Fig. 1). Furthermore, the power analysis curve confirmed that the study’s sample size was sufficient to detect key predictors, achieving a high statistical power of 0.9 or higher (Fig. 2).

**Table 3:** Selected features ranked by SHAP Importance.

Features	Description	SHAP Value
anti_ccp	Biomarker measured through laboratory testing	1.943
tender_j_c	Count of tender joints in clinical examination	1.428
swollen_j_c	Count of swollen joints	0.717
Fatigue	Fatigue	0.170
Digestive	Gastrointestinal manifestations and symptoms	0.145
Age	Age	0.099
Rf	Biomarker measured through laboratory testing	0.095
J-swelling	Joint swelling	0.089
Numbness	Numbness	0.072
Hearing	Hearing impairments and disorders	0.070

The mean values with 95% confidence intervals for the key quantitative variables are also reported in Table 4.

**Table 4:** Mean values with 95% confidence intervals for key quantitative variables in the Study.

Features	Mean With Confidence Interval
Anti_ccp	X̄ = 124.59 ± 28
tender_j_c	X̄ = 4.9 ± 0.4
Swollen_j_c	X̄ = 2 ± 0.3

After determining SHAP values and feature importance, the selected model was re-run using the.

chosen features, with results presented in Table 5. The model’s superior performance was further.

confirmed through 5-fold cross-validation using the selected features (Table 5).

**Fig. 3:**
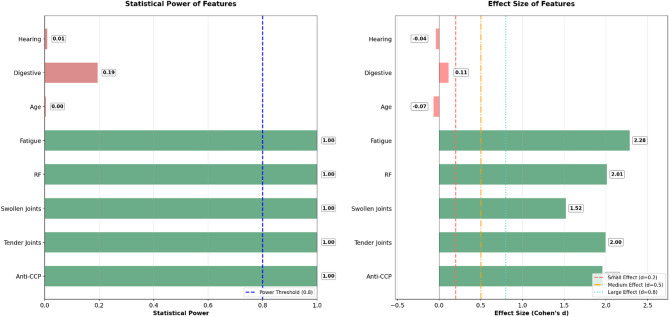
Statistical Power and Effect Size of Key Study Variables.

The ROC curve also demonstrated the improved performance of the CatBoost model after.

incorporating the final selected features (Fig. 3).

**Table 5:** Performance comparison of the selected model: baseline features vs. selected features.

	Metric	Full features	Selected features(By SHAP)
**1**	AUC-ROC	0.954	0.962
**2**	Accuracy	0.921	0.934
**3**	F1-Score	0.927	0.940
**4**	LogLoss	0.220	0.232
**5**	Brier Score	0.060	0.057
**6**	Sensitivity	0.927	0.951
**7**	Specificity	0.914	0.914

**Fig. 4:**
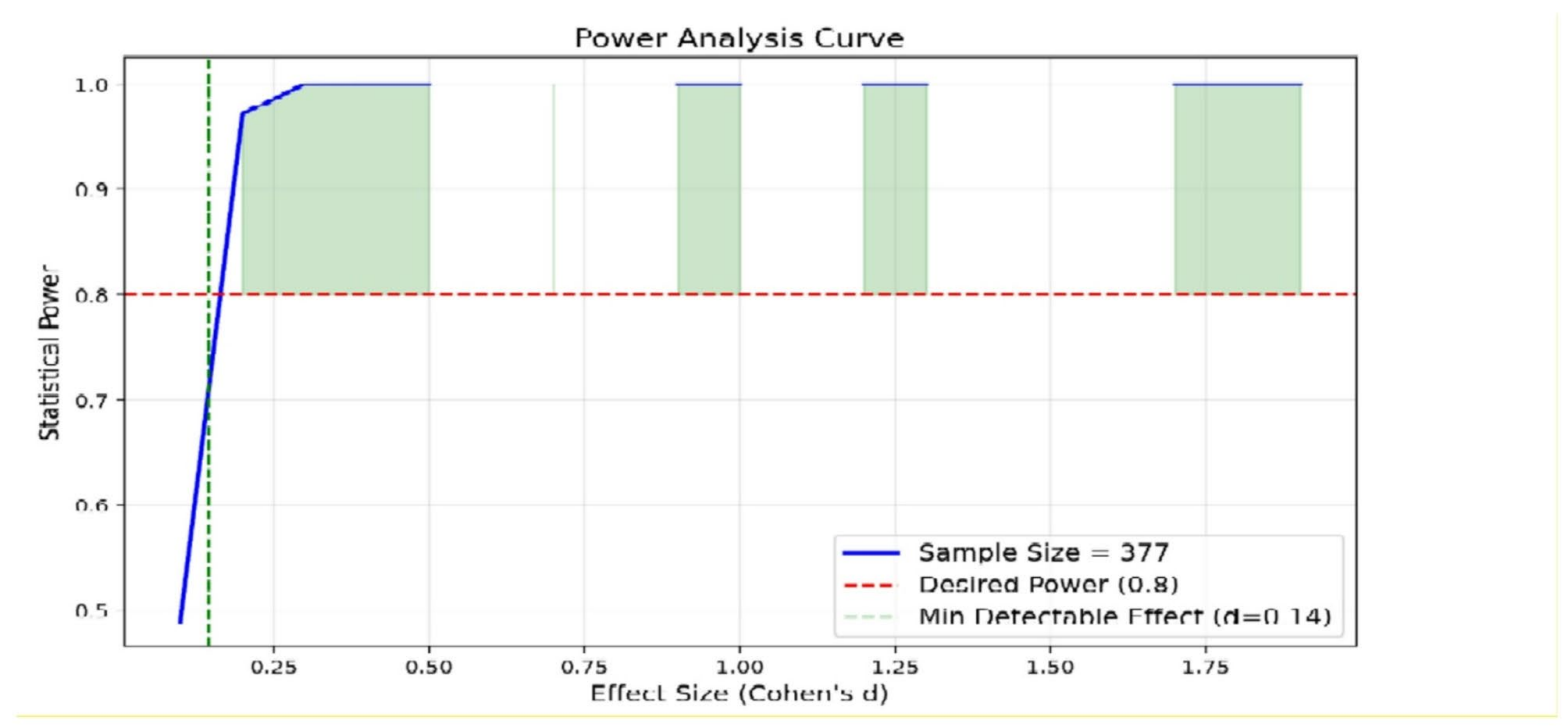
Power Analysis Curve.

In this plot, the vertical axis displays the study variables, and the horizontal axis shows the SHAP values for each feature, illustrating the magnitude and direction of each variable’s impact on the disease prediction probability. Red coloring indicates high feature values, while blue represents low values. When red data points appear on the right side of the plot, the feature has a positive effect on model output; conversely, red points on the left side indicate a negative influence (Fig. 4).

## Discussion

This study identified key factors in diagnosing rheumatoid arthritis, starting with statistical tests. Out of all variables, five—Anti-ccp, tender joint count, swollen joint count, RF, and ESR—were found significant for early detection. Machine learning further analyzed these factors. Results showed that Anti-CCP, tender joint count, swollen joint count, digestive symptoms, fatigue, age, RF, and auditory symptoms were the most important predictors. While Table 3 highlights their role in the final model, a detailed SHAP Plot clarifies how these features influence the output.

When comparing our results with the literature, to the best of our knowledge, no prior study has specifically focused on identifying effective factors for disease diagnosis. However, earlier studies using machine learning methods for disease diagnosis included factors such as pain after rest, morning stiffness, symmetric joint involvement, redness, body pain, joint swelling, history of joint damage, age, sex, and laboratory factors like Anti-ccp, RF, ESR, and Anti-Carp^5,6,8,12,13^.

Anti-CCP antibodies were discovered in 1964 and have been part of the ACR/EULAR classification criteria for RA since 2010^23^. Various studies have emphasized its important role in RA diagnosis^8,24^. The study conducted by Bai et al. aimed to improve disease identification and utilized an artificial neural network, demonstrating that the Anti-CCP factor had the greatest impact on disease detection^8^. In our study, the laboratory factor Anti-ccp showed the highest diagnostic value, with a positive predictive value for the disease. However, since the sensitivity of this test ranges from 41% to 77%^23^, clinical symptoms must also be considered concurrently. Furthermore, RF positivity along with higher anti-CCP levels may improve diagnostic accuracy^24^.

In this study, the tender joint count identified by physical examination and the swollen joint count were significant features in both statistical tests and machine learning methods, demonstrating a positive predictive effect on the model’s output. These factors are also specified in the ACR/EULAR classification criteria for RA. Although Ten Brinck RM et al.^6^, in agreement with our findings, identified these characteristics as diagnostic factors in their study, they had not been used in other modeling research aimed at diagnosing rheumatoid arthritis^5,8,12,13^.

Additionally, studies have shown^25–27^ that fatigue is a common symptom in inflammatory diseases such as rheumatoid arthritis and occurs more frequently in patients than in healthy individuals^28^. Fatigue that does not improve with rest imposes a significant psychological burden on patients because of its substantial impact on quality of life^27^. In line with these studies, our findings also indicated that fatigue was an important factor in disease detection, and, in some cases, its increase was associated with a higher likelihood of RA.

Furthermore, our findings showed that gastrointestinal and auditory issues were also identified as potentially significant factors in disease detection (Table 3). However, according to the SHAP Summary Plot (Fig. 4), these two features had a negative impact on predicting disease probability, meaning that, in our model, higher values of these features decreased the likelihood of RA. These results may reflect the higher prevalence of these symptoms in the control group, as existing research indicates that gastrointestinal manifestations in RA patients typically occur after gastric mucosal changes induced by polypharmacy and long-standing disease^29^. In fact, these two factors explain why rheumatologic patients often develop concurrent gastrointestinal conditions, such as gastric mucosal alterations, gastric atrophy, and intestinal metaplasia^30^. Additionally, one study links Abatacept medication to the formation of intestinal ulcers^31^. While existing studies report gastrointestinal complications in rheumatoid arthritis (RA) patients with long-standing disease who have started disease-modifying antirheumatic drugs (DMARDs), our research focused on early RA diagnosis and initial symptoms, conducted on newly diagnosed patients. As a result, our study found no link between gastrointestinal manifestations and an increased risk of disease progression.

The incidence of hearing impairment is higher in patients with rheumatoid arthritis, with established links to disease activity and duration^32,33^. A study^2^ identifies adverse effects of antirheumatic drugs and decreased microvascular perfusion in the inner and middle ear as factors contributing to hearing impairment in chronic rheumatoid arthritis (RA) patients. Similarly, a study^34^ implicates systemic inflammation in long-standing RA as a cause of auditory problems among patients with established disease. Overall, these findings suggest that hearing deficits are less common during the initial presentation of rheumatoid arthritis.

The variable age was also identified as a significant predictor in our findings, with relatively lower importance, and showed a positive correlation with disease prediction. This finding matches some previous studies^8,35^. While other studies have indicated that sex is a factor in disease occurrence^35,36^, it was not significant in our study. This difference is attributed to the female-majority sample, where 92% of participants were women. As a result, the machine learning models could not distinguish between sexes—male cases were labeled as outliers, making it impossible to determine the significance of female sex in disease detection.

Regarding tobacco use, established as a risk factor in the literature^37^ and examined in our study, this same sampling limitation obscured its role in disease detection. Increasing the sample size could resolve this issue.

Looking toward future advancements, Micro-Electro-Mechanical Systems (MEMS) technology holds great promise for improving the early detection of Rheumatoid Arthritis (RA) in primary care settings. As noted in a study^38^, MEMS sensors enable non-invasive, continuous monitoring of physiological signals related to early RA, such as joint movement patterns and localized temperature changes. Our study, which identifies key clinical and serological predictors, lays the foundation for integration with this technology. In the future, factors identified by our model—such as tender joint count and swollen joint count—could be measured objectively and continuously using MEMS-based wearable devices. This collaboration between machine learning models and real-time MEMS data collection has the potential to transform early screening by providing high-frequency, objective data streams, ultimately enabling earlier and more accurate referrals.

### Study limitations

Despite the promising results, this study has several limitations that should be addressed in future research.

This study was conducted to identify the key factors for detecting rheumatoid arthritis, utilizing data.

collected directly from a real-world clinical setting. This approach, coupled with the strong.

performance of the developed model, underscores the validity of our findings. However, the relatively.

small sample size remains a limitation of this study. Future multi-center studies with larger cohorts.

are recommended to address this limitation.

A gender imbalance was also present in our sample, which is to some extent unavoidable given that.

the disease is more prevalent in women, as established in prior literature. Nevertheless, increasing the.

overall sample size in future work may help achieve a more balanced distribution.

Due to the time constraints of the present study, although robust internal validation using nested cross-validation demonstrated the model’s excellent performance, external validation on an.

independent, multi-center cohort could not be conducted. This critical step, which is essential for.

definitively confirming the model’s robustness and clinical applicability as a screening tool in.

broader populations and diverse healthcare settings, should be undertaken in future studies.

## Conclusion

The results of this study demonstrated that three key factors—Anti-CCP, tender joint count, and swollen joint count—along with other elements such as fatigue, age, and RF, are more effective than other symptoms in identifying rheumatoid arthritis. Given the challenging nature of diagnosing this disease, particularly for general practitioners, these findings can help identify at-risk individuals and facilitate earlier referral to rheumatologists.

## Data Availability

The datasets generated and analyzed during this study are available from the corresponding author on reasonable request via email.
